# Film Mulching Drip Irrigation Improves the Soil Hydrothermal Environment to Enhance Photosynthetic Efficiency and Yield of Sorghum in an Agro-Pastoral Ecotone of Northern China

**DOI:** 10.3390/plants15081157

**Published:** 2026-04-09

**Authors:** Siyu Yan, Wei Xiong, Fengpeng Guo, Baichen Zhang, Jiahao Wang, Matthew Tom Harrison, Ke Liu, Xiaorui Li, Shuqi Dong, Xiangyang Yuan

**Affiliations:** 1 College of Agriculture, Shanxi Agricultural University, Taigu 030801, China; yan18235856798@163.com (S.Y.); xiongwei2366@163.com (W.X.); 19903584888@163.com (F.G.); wjsbsb131548@163.com (B.Z.); 13097583679@163.com (J.W.); lixiaorui@sxau.edu.cn (X.L.); dongshuqi@sxau.edu.cn (S.D.); 2 Tasmanian Institute of Agriculture, University of Tasmania, Newnham Drive, Launceston, TAS 7250, Australia; matthew.harrison@utas.edu.au (M.T.H.); ke.liu@utas.edu.au (K.L.); 3 Special Orphan Crops Research Center of the Loess Plateau, Ministry of Agriculture and Rural Affairs, Shanxi Agricultural University, Jinzhong 030800, China

**Keywords:** agro-pastoral ecotone zone, film mulching drip irrigation, soil hydrothermal environment, photosynthetic efficiency, yield

## Abstract

Film mulching drip irrigation (FMDI) has shown strong yield-promoting effects in arid regions, but its regulatory effects on sorghum, under the unstable soil hydrothermal conditions of the agro-pastoral ecotone zone, remain poorly understood. Sorghum production in this region is frequently constrained by uneven precipitation, high evaporative demand, and limited thermal resources. This study aimed to clarify the role of film mulching drip irrigation in improving the soil hydrothermal environment and photosynthetic performance of sorghum, thereby enhancing yield in the agro-pastoral ecotone of northern China. Compared with bare land without film mulching or drip irrigation (CK), FMDI increased soil temperature by 0.33–2.25 °C and soil moisture by 13.87–18.10% at 0–20 cm depth, alleviating early growth constraints. The leaf chlorophyll b content and carotenoid content of sorghum increased by 55.61% and 55.27%, respectively, while the net photosynthetic rate (Pn) increased by 32.35% and photosystem II (PSII) photochemical efficiency also improved. Random forest (RF) and partial least squares structural equation modeling (PLS–SEM) analyses indicated that chlorophyll, gas exchange, and soil moisture were key drivers of yield formation. Ultimately, FMDI increased yield by 67.08%, indicating that FMDI is an effective irrigation–mulching strategy for improving sustainable sorghum production in the agro-pastoral ecotone zone.

## 1. Introduction

Climate change, global warming, population growth, and water scarcity pose significant challenges to agricultural production [1,2]. Against this backdrop, enhancing crop yields to meet the growing demand for food is a critical issue requiring urgent attention [3,4]. The agro-pastoral ecotone zone is an ecologically fragile region characterized by a predominantly semi-arid climate, serving as a transitional area between semi-arid and semi-humid zones. It is also a vital production area for distinctive dryland agriculture [5]. This region spans vast territories. Taking China’s northern agro-pastoral ecotone as an example, its cultivated land covers approximately 22.93 million ha, accounting for 17.03% of the nation’s total arable land. It serves as a vital base for producing agricultural and livestock products and functions as an ecological security barrier [6,7]. Sorghum (*Sorghum bicolor* L.), the world’s fifth-largest cereal crop, is a staple food for over 500 million people [8,9]. Its low input requirements and versatile applications make it a key crop in this region. Although sorghum is relatively drought tolerant and adaptable to poor soils, its production in the agro-pastoral ecotone zone is still constrained by uneven precipitation, limited thermal resources, large diurnal temperature fluctuations, and intense solar radiation. These conditions frequently expose sorghum to combined water and temperature stress, thereby restricting growth and yield formation [10,11]. Therefore, developing efficient irrigation and cultivation strategies in this region to improve the soil hydrothermal environment and better meet sorghum growth requirements is crucial for mitigating yield uncertainties caused by climate fluctuations. This represents not only an effective pathway to overcome resources and environmental constraints and achieve stable sorghum production and increased yields, but also an urgent necessity for safeguarding food security and promoting sustainable agricultural development in the agro-pastoral ecotone zone.

Drip irrigation and film mulching are commonly used methods to increase soil temperature and moisture content [12,13]. As a precision irrigation technique, drip irrigation delivers water directly and promptly to the crop root zone through a piping system [14,15]. This method effectively reduces water loss from surface evaporation and deep percolation, maintaining root-zone soil moisture within optimal ranges for crop growth and creating favorable hydrological conditions for improving yield [16,17]. Reports indicate that drip irrigation conserves approximately 40–50% more water compared to traditional flood irrigation [18]. Film mulching reduces heat exchange by suppressing air movement at the soil surface, effectively lowering soil moisture evaporation and enhancing water retention capacity [19,20]. Studies indicate that film mulching significantly increases soil moisture content at the 0–20 cm depth, thereby promoting crop growth and improving yield and water productivity [21,22]. Simultaneously, the pronounced warming effect during the early growth stage promotes uniform and rapid seed germination, advances crop phenology, and stimulates canopy development [23,24,25]. Notably, film mulching may cause root and leaf senescence in the later growth stages due to excessive soil temperature elevation, ultimately reducing crop yields [26,27]. In the late 1990s, the integrated technology of FMDI was applied in cotton production in Xinjiang, China. This approach not only retained the temperature-increasing and evaporation-reducing effects of mulching but also provided uniform, regular, and quantified water supply through drip irrigation. Moreover, drip irrigation mitigated the high-temperature stress caused by mulching during the late growth stage, while helping to maintain more favorable root-zone moisture conditions and contributing to yield enhancement [28,29,30]. Presently, the yield-enhancing effects of FMDI have also been validated in wheat and maize production within arid regions. Relevant reports indicate that this technology increased maize and wheat yields in dry areas by 47.3% and 23.6%, respectively [31,32]. However, existing research on FMDI primarily focuses on typical arid regions, and its application effectiveness in the ecologically more complex agro-pastoral ecotone zone remains unclear. Particularly influenced by the transitional climate characteristics of this region, whether this technology can effectively mitigate the constraints from insufficient accumulated temperature and seasonal drought, along with its specific regulatory pathways on sorghum yield formation, requires further validation. Therefore, elucidating the yield-enhancing effects of changes in the soil hydrothermal environment under this technology holds significant guiding importance for boosting of the dryland agricultural production potential in this region and optimizing local sorghum cultivation management.

Crop yield represents the ultimate outcome of growth and development, while photosynthesis provides the material foundation for yield formation [3,33,34]. Research indicates that approximately 90–95% of dry matter originates from photosynthetic products [35,36]. Crop photosynthesis is influenced not only by genetic variation but also by root-zone soil moisture and temperature conditions [37,38]. The yield-regulating effect of film mulching drip irrigation fundamentally operates by altering root-zone soil moisture and temperature, thereby influencing photosynthetic physiological processes. Therefore, deciphering the driving mechanisms of soil hydrothermal environmental changes in sorghum photosynthesis is key to understanding yield formation. Clarifying this physiological regulation process not only explains yield increases at the mechanistic level but also provides crucial theoretical support for optimizing FMDI technology systems in the agro-pastoral ecotone zone.

Accordingly, this study aimed to determine whether film mulching drip irrigation can alleviate the combined challenges of seasonal drought and limited thermal resources in the agro-pastoral ecotone of northern China, and to clarify how its regulation of the soil hydrothermal environment enhances sorghum photosynthetic performance and yield formation.

## 2. Results

### 2.1. Soil Temperature and Moisture Content

**Figure 1:**
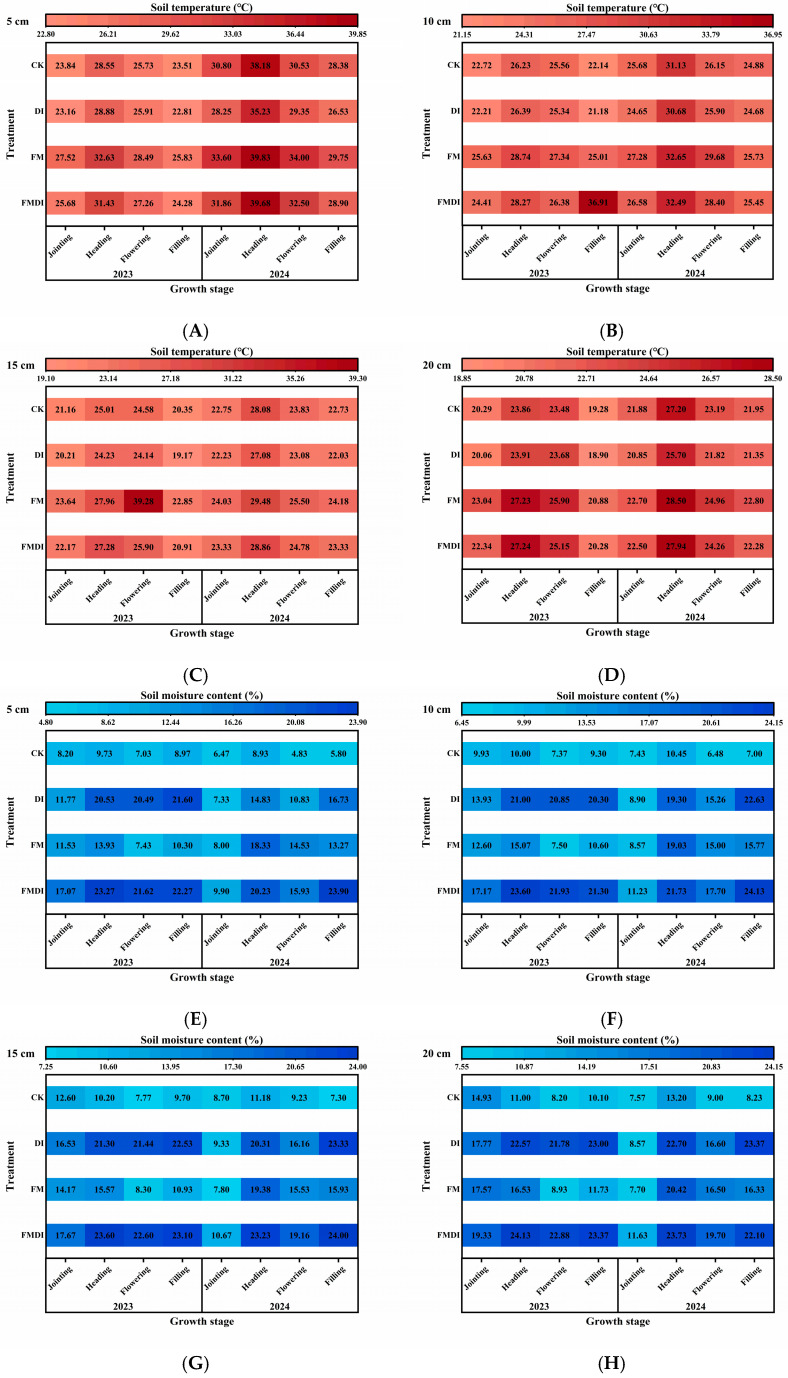
Comparison of soil temperature at 5 cm (**A**), 10 cm (**B**), 15 cm (**C**), and 20 cm (**D**) depth, and soil moisture content at 5 cm (**E**), 10 cm (**F**), 15 cm (**G**), and 20 cm (**H**) depth during the jointing, heading, flowering, and filling stages of sorghum under different treatments in 2023 and 2024. Values shown represent the average of five replicates.

The four treatments were film mulching drip irrigation (FMDI), film mulching without drip irrigation (FM), drip irrigation without film mulching (DI), and bare land without film mulching and drip irrigation (CK). During the four growth stages, soil temperatures at the 0–20 cm depth exhibited similar seasonal variation patterns (Figure 1A–D), following a single-peak curve with an initial rise followed by a decline, peaking at the heading stage. The influence of different treatments on soil temperature was ranked as follows: FM > FMDI > CK > DI. Soil temperature differences among treatments gradually diminished as sorghum matured. At heading, the 5 cm depth soil temperature in the FMDI treatment was –0.15 °C, 4.45 °C, and 1.5 °C higher than in the FM, DI, and CK treatments, respectively. By the grain-filling stage, these differences narrowed to –0.85 °C, 2.37 °C, and 0.52 °C. Differences also decreased with increasing soil depth. At the heading stage, the 5 cm soil temperature difference between FMDI and FM, DI, and CK were 0.15 °C, 4.45 °C, and 1.5 °C, respectively. At the 20 cm depth, differences narrowed to 0.56 °C, 2.24 °C, and 0.74 °C.

Significant differences in soil moisture content were observed among treatments (Figure 1E–H), with the overall pattern being FMDI > DI > FM > CK. FMDI maintained the highest soil moisture content at the 0–20 cm soil depth across all four growth stages. During the jointing stage with relatively concentrated rainfall, no significant differences were observed among FMDI, FM, and DI treatments. However, during the grain-filling stage with reduced rainfall, the 0–20 cm soil moisture content in the FMDI treatment was 18.10%, 17.13%, 16.70%, and 13.87% higher than that in the CK treatment, respectively. Compared to the FM treatment, it increased by 10.63%, 8.36%, 8.07%, and 5.77%, respectively, and compared to the DI treatment, it was higher by 7.17%, 1.50%, and 0.67% during the first three stages, but 1.27% lower at the filling stage.

### 2.2. Agronomic Traits

Under different treatments, significant differences were observed in sorghum plant height (Table S1), stem diameter (Table S2), and leaf area (Table 1). In both years, the highest values were recorded under the FMDI treatment, with the order being FMDI > DI > FM > CK. Plant height and leaf area were higher in 2023 than in 2024, while stem diameter was slightly higher in 2024 than in 2023. During the filling stage, plant height under FMDI, FM, and DI treatments was 34.70%, 16.28%, and 27.39% higher than CK, respectively; stem diameter was 52.52%, 16.32%, and 32.64% higher; and leaf area was 65.94%, 15.97%, and 36.83% higher, respectively.

During the two-year trials, the above-ground dry weight of sorghum (Table 2) gradually increased as the growth stage progressed, reaching its maximum during the filling stage. Under all treatments, dry weight followed the pattern: FMDI > DI > FM > CK. During the 2024 sorghum grain-filling stage, the above-ground dry weight under FMDI, FM, and DI treatments was 197.93%, 39.41%, and 59.09% higher than that under the CK treatment, respectively.

### 2.3. Chlorophyll Content

**Figure 2:**
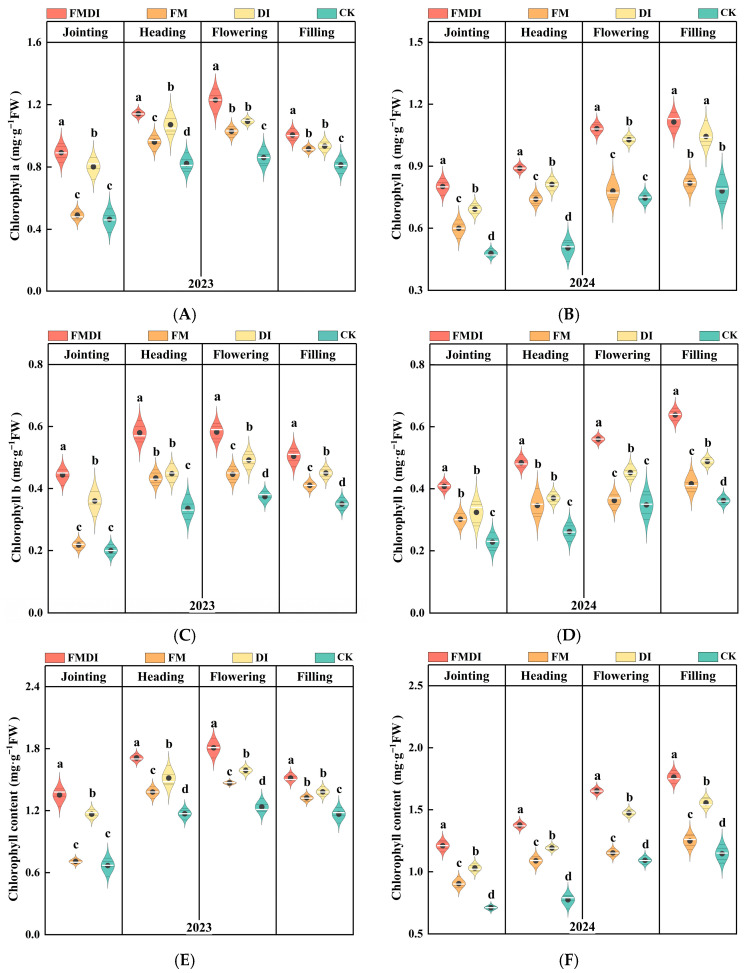

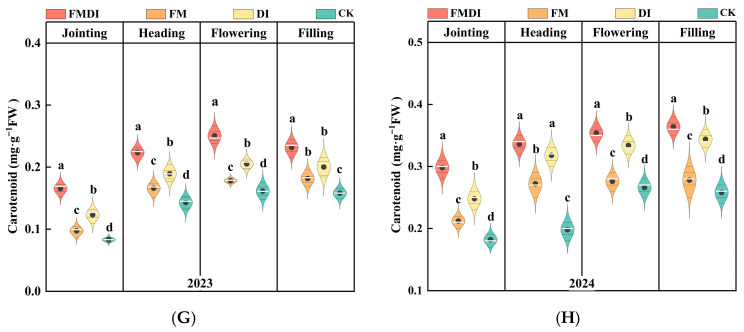
Comparison of chlorophyll a (**A**,**B**), chlorophyll b (**C**,**D**), total chlorophyll (**E**,**F**), and carotenoids (**G**,**H**) during the jointing, heading, flowering, and filling stages of sorghum under different treatments in 2023 (**left column**) and 2024 (**right column**). Different letters within the same year denote significant differences between treatments (*p* < 0.05).

In 2023, sorghum chlorophyll content showed an initial increase followed by a decline, peaking during the flowering stage (Figure 2A,C,E,G). In 2024, it exhibited a gradual upward trend, reaching its maximum during the grain-filling stage (Figure 2B,D,F,H). The FMDI treatment demonstrated significant advantages across all growth stages. During flowering, chlorophyll a content under FMDI treatment was 19.65%, 12.43%, and 43.23% higher than the FM, DI, and CK treatments, respectively; chlorophyll b was 30.49%, 18.29%, and 55.61% higher; and carotenoids were 40.63%, 22.12%, and 55.27% higher. Compared with FM and DI treatments, the DI treatment had a greater effect on the chlorophyll content of sorghum leaves than the FM treatment.

### 2.4. Gas Exchange Parameters

**Figure 3:**
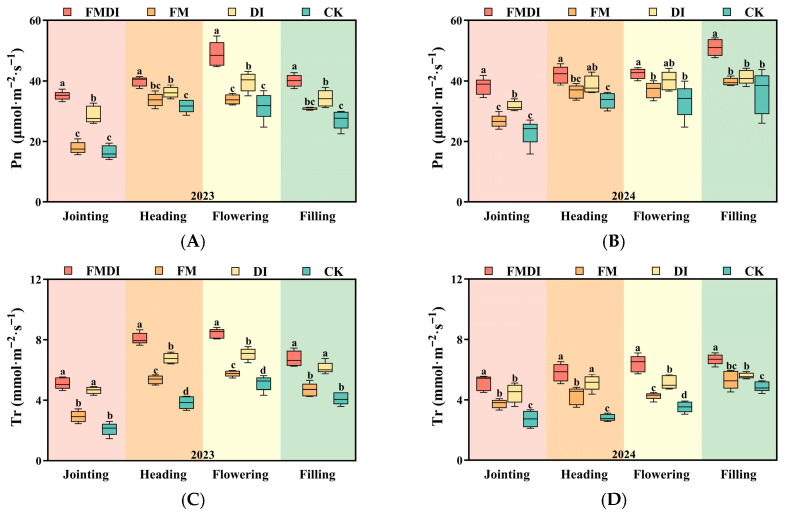

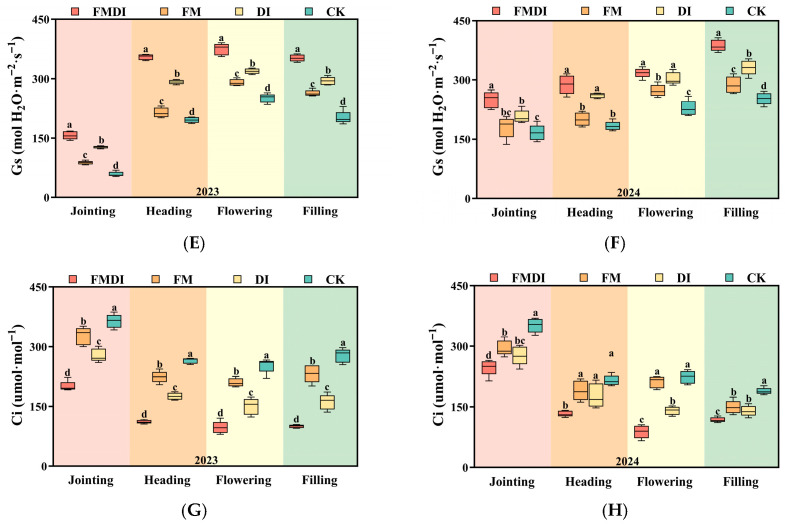
Comparison of gas exchange parameters Pn (**A**,**B**), Tr (**C**,**D**), Gs (**E**,**F**), and Ci (**G**,**H**) during the jointing, heading, flowering, and filling stages of sorghum under different treatments in 2023 (**left column**) and 2024 (**right column**). Different letters within the same year denote significant differences between treatments (*p* < 0.05). Error bars denote standard errors of the means.

Figure 3 shows that film mulching and drip irrigation methods significantly influenced the gas exchange traits of sorghum during critical growth stages, including net photosynthetic rate (Pn), transpiration rate (Tr), stomatal conductance (Gs), and intercellular CO_2_ concentration (Ci). In 2023, gas exchange parameters first increased and then decreased with growth stages, peaking during flowering. In 2024, parameters gradually increased throughout growth stages, reaching maximum values during grain filling. Compared to CK, Pn values increased by 32.35%, 3.30%, and 5.99% for FMDI, FM, and DI, respectively, during grain filling; Tr increased by 38.05%, 12.47%, and 15.59%, respectively; Gs increased by 55.36%, 15.06%, and 33.00%, respectively; while Ci decreased by 59.14%, 26.16%, and 33.44%, respectively.

### 2.5. Chlorophyll Fluorescence Parameters

**Figure 4:**
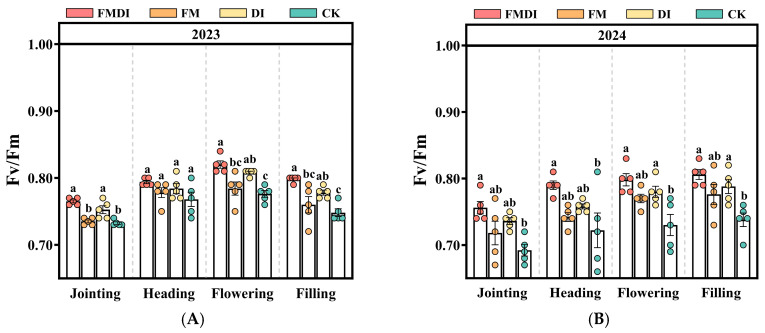

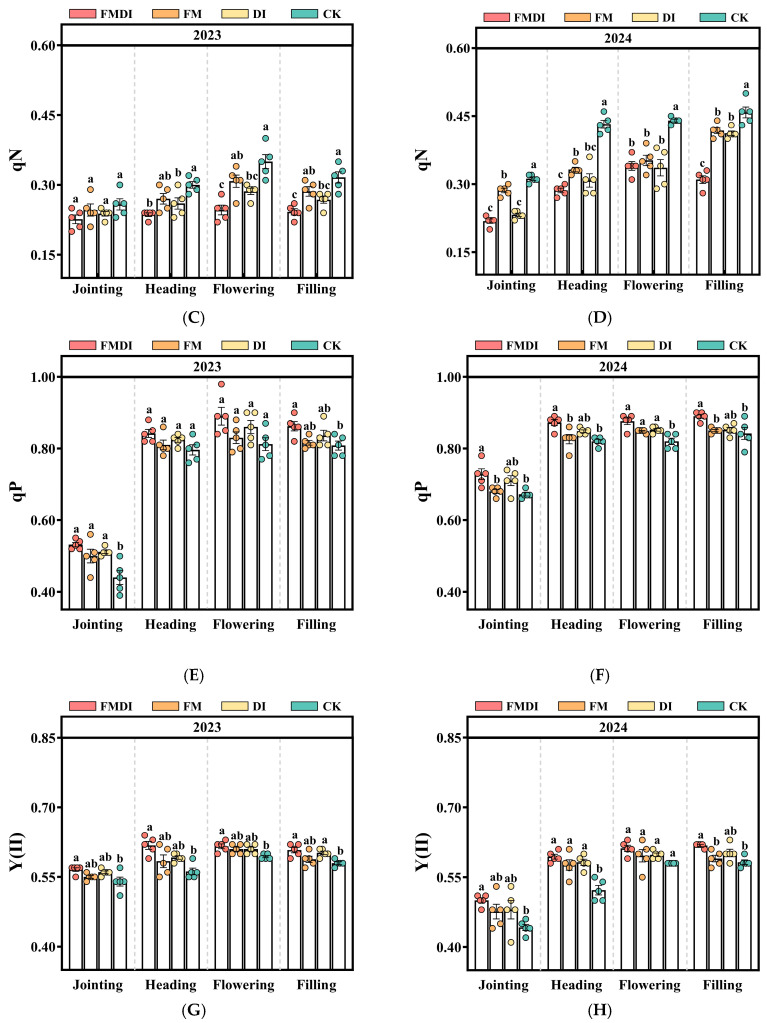
Comparison of chlorophyll fluorescence parameters Fv/Fm (**A**,**B**), qN (**C**,**D**), qP (**E**,**F**), and Y(II) (**G**,**H**) during the jointing, heading, flowering, and filling stages of sorghum under different treatments in 2023 (**left column**) and 2024 (**right column**). Different letters within the same year indicate significant differences among treatments within that growing season (*p* < 0.05). Circles represent data points (*n* = 5). Error bars denote standard errors of the means.

The maximum quantum efficiency of PSII photochemistry (Fv/Fm) showed an initial increase followed by a decline from the jointing stage to the filling stage, peaking during flowering (Figure 4A,B). Compared to CK, FMDI, FM, and DI increased Fv/Fm by 9.46%, 5.41%, and 6.76%, respectively, during grain filling. Similar improvements were observed for the photochemical quenching coefficient (qP) (Figure 4E,F) and the effective quantum yield of PSII photochemistry (Y(II)) (Figure 4G,H). In contrast, the non-photochemical quenching coefficient (qN) (Figure 4C,D) was highest under CK and lowest under FMDI, with FMDI, FM, and DI reducing qN by 48.39%, 9.52%, and 12.20%, respectively, compared with CK.

### 2.6. Yield Components and Yield

Film mulching and drip irrigation methods positively influenced yield traits over two years, enhancing sorghum ear length, ear width, ear weight, grain weight per ear, thousand-grain weight, and overall yield (Table 3). Under FMDI, FM, and DI treatments, sorghum yield increased by 67.08%, 9.52%, and 34.67%, respectively, compared to the CK control. Similar improvements were observed in ear length, ear width, spike weight, grain weight per spike, and thousand-grain weight.

### 2.7. PLS–SEM Structural Equation Modeling and RF Analysis

**Figure 5:**
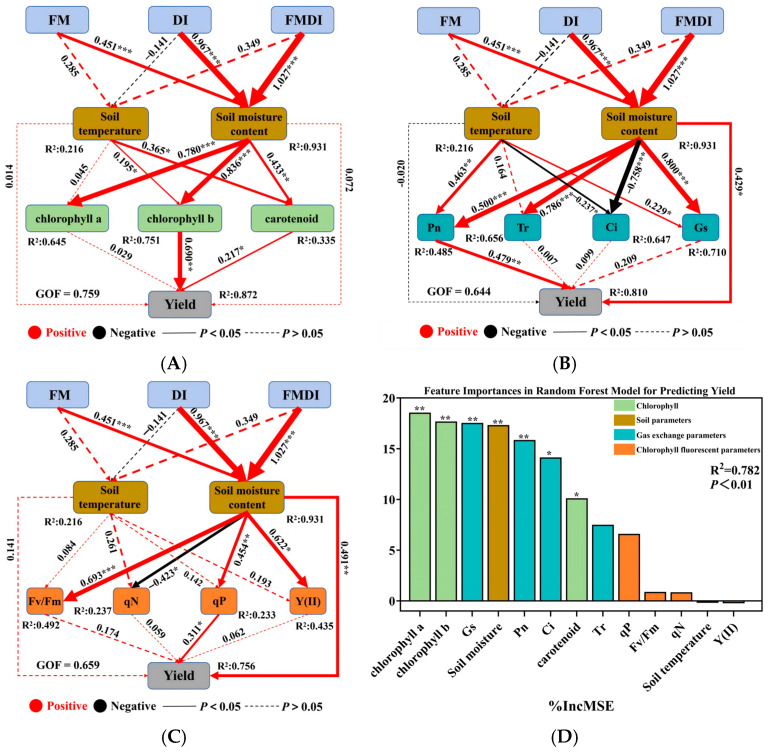
RF and PLS-SEM analyses illustrating the effects of irrigation and mulching practices on sorghum yield. Panel (**D**) shows the relative importance of soil hydrothermal variables (soil temperature and soil moisture) and physiological traits for yield prediction based on the RF model. Panels (**A**–**C**) present PLS-SEM results depicting the indirect effects of FMDI, FM, and DI on sorghum yield mediated by soil hydrothermal conditions and subsequently by (**A**) photosynthetic pigments, (**B**) gas exchange parameters, and (**C**) chlorophyll fluorescence parameters. Numbers on the arrows represent standardized path coefficients, and values within boxes indicate coefficients of determination (R^2^) for endogenous variables. * indicates *p *< 0.05, ** indicates *p* < 0.01, and *** indicates *p* < 0.001.

To further elucidate the yield formation mechanism of sorghum in the agro-pastoral ecotone zone, this study employed the RF algorithm to rank the importance of 13 key environmental and physiological factors affecting yield (Figure 5D). Results indicate that seven key indicators among environmental factors and photosynthetic physiological characteristics are the primary drivers of sorghum yield variation. In descending order of importance: chlorophyll a (18.56%), chlorophyll b (17.69%), Gs (17.55%), soil moisture content (17.33%), Pn (15.86%), Ci (14.14%), carotenoids (10.12%), and Tr (7.50%). Three PLS–SEM were constructed to quantify the causal pathways regulating yield through photosynthetic pigments, gas exchange parameters, and chlorophyll fluorescence parameters. Model fitting results showed all three models possessed extremely high explanatory power, with goodness of fit (GOF) values of 0.759, 0.644, and 0.659, respectively, indicating the models effectively reflected the intrinsic relationships among variables. As shown in Figure 5A–C, FMDI, FM, and DI treatments significantly increased soil moisture content, with FMDI exerting the strongest effect (β = 1.027). FM and FMDI treatments elevated soil temperature. The improved soil hydrothermal environment significantly promoted the accumulation of chlorophyll a (β = 0.780), chlorophyll b (β = 0.836; β = 0.195), and carotenoids (β = 0.433; β = 0.365). Subsequently, chlorophyll b (β = 0.690) and carotenoids (β = 0.217) acted as key mediating variables, exerting significant positive direct effects on yield. In the gas exchange parameter model, improved soil hydrothermal environment conditions significantly positively regulated Pn (β = 0.500; β = 0.463), Tr (β = 0.786), and Gs (β = 0.800; β = 0.229), while exhibiting a significant negative correlation with Ci (β = –0.758; β = –0.237). Among these, Pn (β = 0.479) made the largest direct contribution to yield. In the chlorophyll fluorescence parameter model, increased soil moisture significantly positively regulated Fv/Fm (β = 0.693), qP (β = 0.454), and Y(II) (β = 0.622), with qP being the key factor driving yield increase (β = 0.311).

## 3. Discussion

### 3.1. Soil Temperature and Moisture Content

The soil hydrothermal environment constitutes one of the core factors limiting crop productivity in arid regions and the agro-pastoral ecotone zone. It directly influences root vitality, nutrient uptake efficiency, and photosynthetic potential throughout the growing season, serving as the primary environmental foundation for crop yield formation [39]. Film mulching and drip irrigation can modify root-zone hydrothermal conditions by suppressing direct soil evaporation and reducing rapid fluctuations in soil–atmosphere heat exchange, thereby helping maintain higher soil moisture and a more stable soil temperature, especially during the early and middle growth stages [40,41,42]. In the agro-pastoral ecotone zone, insufficient thermal resources during the early growth stage can restrict sorghum establishment, while soil evaporation still occurs under strong radiation and dry atmospheric conditions; therefore, improving both soil moisture retention and thermal stability is important for early crop growth. Previous studies have confirmed that in maize, wheat, and cotton production across arid and semi-arid regions, FMDI improves the soil hydrothermal environment, thereby enhancing yields [43,44,45]. In this study, FMDI similarly exhibited significant warming and humidifying effects in the agro-pastoral ecotone zone with transitional climatic characteristics. Compared to CK, FMDI increased 0–20 cm soil temperature by an average of 0.33–2.25 °C and raised soil moisture content by 13.87–18.10%. This occurs because FMDI impedes moisture exchange between the atmosphere and soil, suppressing soil evaporation and thereby reducing heat exchange and latent heat flux between soil and air [46,47]. Simultaneously, it traps solar energy, preventing solar heat from radiating from the soil surface into the surrounding air [48,49], thus elevating soil temperature and moisture content. Furthermore, this study found that soil temperature differences among treatments gradually diminished as the growing season progressed, consistent with previous research on sweet potato and corn crops [50,51]. In later growth stages, as the plastic mulch became covered by dense foliage, the soil temperature difference between mulched and unmulched treatments was no longer significant. This may occur because the larger canopy in the later growth stage reduces solar radiation reaching the ground, thereby suppressing the influence of the plastic mulch’s optical properties on soil temperature [52,53]. Simultaneously, in reports on plastic mulch application in arid regions, researchers have found that high soil temperatures under FM during the later crop-growth stage stimulate crown and root senescence, reduce nutrient absorption capacity and crop photoperiod synchronization rates, shorten the reproductive growth period, and decrease crop productivity [54,55]. In this study, we also observed that FM produced a greater warming effect than FMDI, yet no premature senescence occurred. This may be attributed to the agro-pastoral ecotone zone’s characteristics: low annual average temperatures, significant diurnal temperature fluctuations, and a relatively low probability of extreme summer heat. These conditions allow the warming effect of FM to compensate for the lack of thermal resources during the early-to-mid growth stages. Additionally, the region’s relatively low air humidity and high wind speeds facilitate heat dissipation from the canopy and soil surface. Further analysis using PLS–SEM revealed that soil moisture exerted a greater influence than soil temperature on photosynthetic pigments, gas exchange, and fluorescence characteristics across all path models. RF ranking also confirmed soil moisture’s higher importance relative to soil temperature. Unlike studies conducted in typical arid regions, our results indicate that under the agro-pastoral ecotone context, soil moisture played a stronger regulatory role than soil temperature in shaping sorghum’s photosynthetic performance and yield, although moderate warming remained important during early growth.

### 3.2. Chlorophyll Content and Photosynthetic Characteristics

Photosynthesis serves as the foundation for crop growth, development, and yield formation, constituting the fundamental source for the synthesis, accumulation, and energy acquisition of photosynthetic products [20,56]. Chlorophyll, as the light-harvesting pigment in photosynthesis, is a crucial indicator for assessing crop photosynthetic potential and physiological status. Therefore, maintaining high chlorophyll levels provides the material basis for ensuring high photosynthetic efficiency and high yields [57]. The importance ranking results from the RF algorithm also indicate that chlorophyll-related indicators contribute significantly to yield among all physiological factors. This study found that chlorophyll a, b, and carotenoids were significantly higher in the FMDI treatment than in other treatments at all growth stages. Although chlorophyll synthesis is primarily regulated by endogenous genes, environmental factors also play a crucial role [58]. This was further validated by the PLS–SEM model, where the improved soil hydrothermal environment under FMDI treatment significantly positively influenced chlorophyll content. This effect likely stems from FMDI enhancing soil hydrothermal conditions, thereby boosting root activity and improving the root system’s capacity to absorb water and nutrients [46,59]. Concurrently, a larger canopy area enhances light capture, which promotes chlorophyll synthesis within leaves [60]. Furthermore, our study revealed that chlorophyll b and carotenoids exerted a stronger positive regulatory effect on yield formation than chlorophyll a. This finding is closely linked to the unique climatic conditions of the agro-pastoral ecotone zone, characterized by high radiation and low accumulated temperature. Chlorophyll b broadens the light absorption spectrum and enhances the efficiency of light energy transfer to chlorophyll a [61], while carotenoids protect PSII reaction centers from photoinhibition damage under intense radiation [62]. Higher chlorophyll b and carotenoid levels help maintain photosystem stability and delay leaf senescence in later growth stages, thereby ensuring more efficient photosynthetic assimilation and dry matter accumulation during filling [63,64].

Gas exchange parameters serve as key physiological indicators reflecting plant photosynthetic and metabolic rates [65]. Chlorophyll fluorescence parameters are a crucial tool for evaluating PSII energy allocation and photochemical performance, thereby assessing crop light utilization capacity [66]. In this study, compared to the CK, FMDI treatment significantly increased Pn, Tr, and Gs across all growth stages of sorghum while simultaneously reducing Ci. This occurs because FMDI provides sorghum with ample water supply, promoting stomatal opening and enhancing CO_2_ and H_2_O exchange capacity, thereby enabling efficient photosynthetic carbon assimilation [67,68]. However, Ci does not simply reflect stomatal opening alone, but rather the dynamic balance between stomatal CO_2_ supply and mesophyll CO_2_ consumption. Although stomatal opening increases CO_2_ diffusion into the leaf, the stronger carbon assimilation demand under FMDI can consume CO_2_ more rapidly in the mesophyll than it is replenished through stomatal diffusion, resulting in a lower Ci despite a higher Gs [69]. Therefore, the simultaneous increase in Gs and decrease in Ci observed in this study suggests that FMDI enhanced not only stomatal conductance, but also the biochemical capacity for CO_2_ fixation, thereby strengthening overall photosynthetic performance. This aligns with the results from RF ranking and PLS–SEM modeling, indicating that FMDI improves the soil hydrothermal environment, enhances leaf gas exchange capacity and carbon assimilation rates, thereby providing a crucial physiological basis for its yield-enhancing effect in the agro-pastoral ecotone zone. Concurrently, enhanced photosynthetic efficiency allows leaves to utilize light energy more effectively. To sustain elevated carbon assimilation fluxes, PSII must adjust its photochemical efficiency, enabling the light reaction to supply sufficient energy for the intensified assimilation process [63,70]. In this study, FMDI significantly increased Fv/Fm, qP, and Y(II), indicating enhanced maximum PSII photochemical efficiency. More reaction centers remained open, improving electron transport efficiency, and thereby more effectively driving photosynthetic carbon assimilation [47,71]. Furthermore, the reduction in qN indicates decreased non-photochemical energy dissipation. Leaves reduced energy loss through thermal dissipation, allowing more excitation energy to be directed toward photochemical reactions, resulting in a significant improvement in light energy conversion efficiency under FMDI [72]. In the PLS–SEM model, qP exhibits a significant positive effect on yield, further demonstrating that the higher photochemical quenching capacity in PSII promotes photosynthetic carbon assimilation and ultimately yield formation by enhancing electron transport efficiency and light energy utilization efficiency.

### 3.3. Agronomic Traits and Yield

Agronomic traits and yield serve as core indicators for evaluating the practical value of cultivation techniques [73]. Superior agronomic traits form the morphological foundation for high crop yields [64]. In this study, the FMDI treatment enhanced sorghum plant height, stem thickness, leaf area, and dry matter yield. This improvement primarily resulted from the early warming effect of FMDI, which accelerated sorghum growth and development while strengthening resistance to early frost and low temperatures [74]. Stable water supply in the later growth stage significantly increased chlorophyll content, gas exchange capacity, and PSII photochemical efficiency, laying the foundation for grain filling and yield formation [72]. Results indicate that compared to CK, FMDI increased sorghum yield by 67.08% in the agro-pastoral ecotone zone, consistent with findings from related studies on maize production in arid regions [75]. Considering FMDI’s remarkable water–heat regulation and stable yield enhancement capabilities under the unique climatic conditions of the agro-pastoral ecotone zone, we conclude that FMDI is an effective irrigation–mulching strategy for improving the soil hydrothermal environment and enhancing sorghum production in this region.

## 4. Materials and Methods

This experiment was conducted from April 2023 to October 2024 in Shanyin County, Shuozhou City, Shanxi Province (39°31′33.2″ N, 112°48′35.3″ E). This region is a typical agro-pastoral ecotone zone, situated at an elevation of 1002 m, with a frost-free period of 130 days annually, and an average annual precipitation of 410 mm. The preceding crop, for both 2023 and 2024, was millet. Two years of field trials were conducted at the same experimental station and in the same experimental plot. Before sowing each year and prior to treatment establishment, five soil cores from the 0–20 cm layer were collected randomly across the experimental field, mixed thoroughly to form one composite sample, and used for the determination of soil nutrient content, shown in Table 4.

### 4.1. Experimental Design

**Figure 6:**
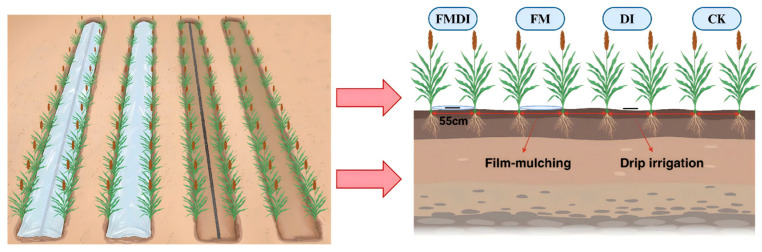
Diagram of treatments; FMDI: film mulching drip irrigation, FM: film mulching without drip irrigation, DI: drip irrigation without film mulching, CK: bare land without film mulching or drip irrigation.

**Figure 7:**
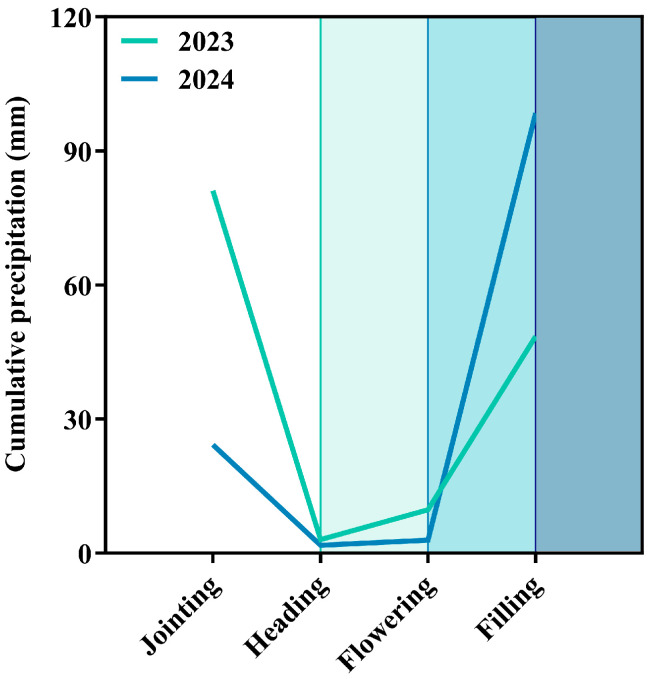
Cumulative precipitation during the jointing, heading, flowering, and filling stages of the Jinza 22 sorghum variety in 2023 and 2024.

This experiment employed a completely randomized block design with four treatments: film mulching drip irrigation (FMDI), film mulching without drip irrigation (FM), drip irrigation without film mulching (DI), and bare ground without film mulching or drip irrigation (CK), each replicated five times. The treatment layout is shown in Figure 6. Each experimental plot measured 60 m long and 4.4 m wide (area: 264 m^2^), comprising eight rows with 55 cm row spacing. The experimental sorghum variety was Jinza 22, sown on 26 April 2023, and 26 April 2024. Prior to sowing, the field was rotary tilled and plowed. Cow manure and a stabilized controlled–release compound fertilizer (total N-P_2_O_5_-K_2_O ≥ 51%) were applied as basal fertilizer, with no additional topdressing throughout the growing season. Irrigation treatments (FMDI and DI) were applied via single-wing drip tape during the jointing, heading, and flowering stages, with each irrigation delivering 30 mm of water. The non-irrigated treatment relied solely on natural precipitation. Phenological stages were monitored by regular field observation throughout the growing season, and each growth stage was recorded when the majority of plants in a plot had reached the corresponding developmental stage. The jointing, heading, flowering, and grain-filling stages were observed on July 1, August 14, August 18, and September 3 in 2023, and on June 28, August 3, August 8, and August 23 in 2024, respectively. Figure 7 shows the cumulative precipitation for each period. Field management practices, including weed control and pest/disease management, followed standard local agricultural protocols. Sorghum was harvested on October 8 2023, and October 4 2024. All field operations were performed by the same individual in both years to ensure the consistency of experimental conditions.

### 4.2. Sample Collection and Data Analysis

#### 4.2.1. Soil Temperature and Moisture Content

Using bent-tube soil temperature probes, daily temperature variations were measured at soil depths of 5 cm, 10 cm, 15 cm, and 20 cm for each treatment during the sorghum’s jointing stage, heading stage, flowering stage, and filling stage. Measurements were taken at 8:00, 11:00, 15:00, and 18:00, with five replicates per treatment [39].

Using a soil moisture meter, the moisture content at soil depths of 5 cm, 10 cm, 15 cm and 20 cm was measured for each treatment during the sorghum’s jointing stage, heading stage, flowering stage, and grain-filling stage. Each treatment was replicated five times [74].

#### 4.2.2. Agronomic Traits

##### Plant Height, Stem Thickness, and Leaf Area

During the jointing, heading, flowering, and filling stages of sorghum, randomly select 10 representative plants with consistent growth from each treatment as samples. After removing the root system, measure plant height using a ruler, and measure stem thickness and the length and width of the second leaf using a vernier caliper [76]. Calculate leaf area using the following formula:

(1) $$Leaf\;area\;({cm}^{2})=leaf\;length\;(cm)\times leaf\;width\;(cm)\times 0.75$$

##### Above-Ground Dry Weight

Use scissors to cut the plant at the base where it emerges from the soil (the above-ground portion), then weigh the fresh weight using an electronic analytical balance (Mettler–Toledo, Zurich, Switzerland). Place the weighed fresh-weight above-ground samples into an oven (Shanghai Boxun Industrial Co., Ltd. Shanghai, China) for de–enzyming at 105 °C for 30 min. Then, reduce the temperature to 80 °C and dry to constant weight, weighing the sample’s dry mass.

#### 4.2.3. Chlorophyll Content

Chlorophyll content was determined using the 96% ethanol extraction method. Accurately weigh 0.1 g of the penultimate leaf from fresh sorghum plants, record the fresh weight (FW), and chop it into small pieces before transferring to a 15 mL centrifuge tube. Add 10 mL of 96% ethanol and leave in the dark at room temperature for 24–48 h, shaking occasionally until the leaves turn completely white. This procedure was repeated four times. Using a UV-visible spectrophotometer, we measured the absorbance values of the solution at wavelengths of 470 nm, 649 nm, and 665 nm. Chlorophyll content was calculated according to the formula [77]:

(2) $$Chlorophyll\;a\;(Chl\;a)=13.95\times {\mathrm{OD}}_{665}-6.88\times {\mathrm{OD}}_{649}$$

(3) $$Chlorophyll\;b\;(Chl\;b)=24.96\times {\mathrm{OD}}_{649}-7.32\times {\mathrm{OD}}_{665}$$

(4) $$Carotenoids\;(Car)=(1000\times {\mathrm{OD}}_{470}-2.05\times Chl\;a-114.8\times Chl\;b)/245$$

(5) $$Chlorophyll\;content\;(mg/g\;FW)=C\times VT\times \frac{n}{FW}\times 1000$$

#### 4.2.4. Gas Exchange Parameters

During the jointing, heading, flowering, and grain-filling stages of sorghum, select clear, cloudless days and use a CI–340 portable photosynthesis meter (CID Bio–Science, Camas, WA, USA) between 9:00 AM and 11:00 AM to adjust the airflow to 300–500 μmol/s. During measurements, the photosynthetic photon flux density (PPFD) at the leaf surface was approximately 800–1600 μmol m^−2^ s^−1^, and CO_2_ concentration was maintained at the ambient level. The Pn, Tr, Gs, and Ci of the second-to-last leaf were recorded, and five leaves were measured per plot [78].

#### 4.2.5. Chlorophyll Fluorescence Parameters

Fluorescence parameters were measured using the PAM–2500 portable pulse-modulated chlorophyll fluorescence meter (Heinz Walz GmbH, Effeltrich, Germany) during the jointing, heading, flowering, and filling stages of sorghum. Leaves underwent thorough dark adaptation for over 30 min, and chlorophyll fluorescence parameters were measured in the second-to-last leaf of sorghum plants. Parameters measured included Fv/Fm, Y(II), qP, and qN.

#### 4.2.6. Yield and Yield Components

During the late wax ripening stage of sorghum, a 22 m^2^ area was randomly selected from each treatment plot for actual harvesting. Yield was determined by weighing, while panicle length and width were measured using a ruler and vernier caliper. Thousand-grain weight was measured using a 10,000-gram analytical balance (Mettler–Toledo, LLC. Shanghai, China) to measure spike weight and grain weight per spike. Thousand-grain weight was determined using a seed analyzer (SC–G Automatic Seed Analyzer and Thousand-Grain Weight Tester, Hangzhou Wanshen Testing Technology Co., Ltd. Hangzhou, China). Each treatment was replicated five times.

### 4.3. Statistical Analysis

Data collation and preliminary processing were performed using Microsoft Excel 2021 (Microsoft, Redmond, WA, USA). Analysis of variance (ANOVA) was conducted using IBM SPSS Statistics 23 (SPSS Inc., Chicago, IL, USA). Significant differences in mean values between treatments were tested using Duncan’s new multiple range test at the *p* < 0.05 level. All figures were plotted using Origin 2021 (Origin Lab, Northampton, MA, USA) and Prism 10.4.2.

Random forest (RF) regression analysis using the R language was employed to evaluate and rank the relative importance of each measured physiological indicator in predicting sorghum yield. Variable importance was determined based on the percentage increase in mean squared error (% IncMSE), with model significance analyzed using the A3 package in R. This analysis provides data-driven evidence for identifying the primary drivers of yield formation.

To systematically quantify causal pathways linking management practices, soil hydrothermal environment, crop physiological traits, and yield, PLS–SEM was employed. This analysis was conducted using the plspm package in R. Based on a crop physiology theoretical framework, three independent models were constructed, each corresponding to distinct physiological indicators: (1) photosynthetic pigments (chlorophyll a, b, and carotenoids); (2) gas exchange parameters (Pn, Gs, Ci, and Tr); and (3) chlorophyll fluorescence parameters (Fv/Fm, qP, qN, and Y(II)). Within these models, treatment measures (FMDI, FM, and DI) were set as exogenous variables, while soil conditions and physiological traits served as endogenous mediating variables influencing yield. Path relationships within the structural models were tested using path coefficients (β) and their significance (based on 5000 Bootstrap resamples), with model explanatory power assessed via the coefficient of determination (R^2^). Overall model goodness of fit was assessed using the GOF (goodness of fit) index, defined as the geometric mean of average communality and average R^2^, serving as a comprehensive metric for PLS model performance at both measurement and structural levels. According to the criteria established by Tenenhaus et al., GOF values of 0.10, 0.25, and 0.36 represent a weak, moderate, and strong model fit, respectively [79].

Because the primary objective of this study was to compare treatment effects within each growing season, data were analyzed and presented separately for 2023 and 2024.

## 5. Conclusions

In summary, FMDI significantly improved the root-zone soil hydrothermal environment in the agro-pastoral ecotone zone and promoted sorghum growth and yield formation. FMDI increased chlorophyll content, Pn, and Gs, and improved PSII photochemical performance, as reflected by higher Fv/Fm, Y(II), and qP. More importantly, by integrating RF and PLS–SEM, this study showed that hydrothermal regulation enhanced photosynthetic performance and thereby drove yield improvement, with soil moisture emerging as a particularly important factor. These findings provide region-specific evidence and a mechanistic basis for optimizing sorghum cultivation under the transitional climatic conditions of the agro-pastoral ecotone zone.

## Figures and Tables

**Table 1:** Comparison of sorghum leaf areas at different growth stages and under different treatments in 2023–2024.

Year	Treatment Code	Leaf Area (cm^2^)			
		Jointing	Heading	Flowering	Filling
2023	FMDI	384.54 ± 81.86 a	485.18 ± 34.13 a	508.45 ± 33.88 a	560.75 ± 80.22 a
	FM	281.36 ± 53.85 b	337.31 ± 39.74 bc	370.65 ± 54.50 bc	430.08 ± 8.58 bc
	DI	313.37 ± 61.78 ab	376.44 ± 38.49 b	425.63 ± 104.70 ab	496.72 ± 45.27 ab
	CK	249.84 ± 36.80 b	291.55 ± 58.98 c	295.79 ± 49.34 c	387.35 ± 34.27 c
2024	FMDI	410.48 ± 65.43 a	393.28 ± 49.72 a	537.23 ± 43.19 a	545.93 ± 40.70 a
	FM	276.98 ± 22.96 bc	338.87 ± 93.67 a	339.08 ± 34.04 b	381.55 ± 99.55 b
	DI	300.73 ± 25.57 b	383.07 ± 59.36 a	385.94 ± 31.86 b	450.16 ± 80.66 ab
	CK	226.46 ± 20.33 c	292.10 ± 73.85 a	317.31 ± 84.51 b	329.00 ± 74.01 b

**Note:** Values are means ± SE (*n* = 5). Different letters within the same year indicate significant differences among treatments (*p* < 0.05).

**Table 2:** Comparison of sorghum’s above-ground dry weights at different growth stages and under different treatments in 2023–2024.

Year	Treatment Code	Above-Ground Dry Weight (g)			
		Jointing	Heading	Flowering	Filling
2023	FMDI	32.11 ± 2.34 a	103.65 ± 2.88 a	113.81 ± 6.33 a	168.53 ± 4.97 a
	FM	20.25 ± 0.73 b	65.46 ± 4.28 b	72.87 ± 4.11 b	97.01 ± 1.91 bc
	DI	24.31 ± 1.07 b	71.62 ± 6.73 b	78.75 ± 1.95 b	105.04 ± 0.28 b
	CK	15.17 ± 1.41 c	60.46 ± 4.74 b	67.94 ± 5.70 b	93.43 ± 0.25 c
2024	FMDI	31.09 ± 5.41 a	130.62 ± 17.53 a	139.47 ± 31.93 a	282.77 ± 47.79 a
	FM	16.82 ± 5.69 bc	58.26 ± 5.00 c	82.40 ± 30.11 b	132.31 ± 6.68 bc
	DI	24.32 ± 5.50 ab	93.35 ± 24.42 b	110.26 ± 35.94 ab	150.99 ± 28.05 b
	CK	14.11 ± 2.76 c	46.37 ± 4.15 c	72.45 ± 7.03 b	94.91 ± 17.11 c

**Note:** Values are means ± SE (*n* = 5). Different letters within the same year indicate significant differences among treatments (*p* < 0.05).

**Table 3:** Comparison of sorghum yields and yield components across different treatment groups and time periods in 2023–2024.

Year	Cultivation Mode	Yield and Yield Composition					
		Ear Length	Ear Width	Spike Weight	Grain Weight	Thousand-Grain Weight	Yield
		(cm)	(cm)	(g)	(g)	(g)	(kg ha^−1^)
2023	FMDI	33.60 ± 1.03 a	8.35 ± 0.13 a	137.55 ± 6.74 a	90.65 ± 2.10 a	29.23 ± 0.19 a	8394.64 ± 140.49 a
	FM	28.18 ± 0.97 c	7.10 ± 0.28 b	99.25 ± 4.25 b	66.93 ± 2.04 c	28.51 ± 0.52 b	6356.69 ± 141.57 c
	DI	30.98 ± 0.59 b	7.50 ± 0.42 b	105.15 ± 3.38 b	81.93 ± 2.31 b	29.11 ± 0.16 a	7634.34 ± 897.90 b
	CK	25.00 ± 0.54 d	6.03 ± 0.35 c	73.15 ± 3.96 c	55.75 ± 2.24 d	25.99 ± 0.18 c	5960.30 ± 118.58 c
2024	FMDI	31.88 ± 4.88 a	8.15 ± 1.15 a	133.58 ± 13.50 a	110.83 ± 9.59 a	35.09 ± 0.66 a	10,706.35 ± 700.53 a
	FM	26.75 ± 1.82 b	7.18 ± 0.42 bc	108.10 ± 13.66 b	73.15 ± 8.46 c	33.25 ± 0.51 b	7018.10 ± 493.82 c
	DI	31.08 ± 0.98 a	7.80 ± 1.02 bc	114.23 ± 9.15 b	90.95 ± 7.59 b	34.04 ± 0.52 b	8630.05 ± 320.18 b
	CK	22.53 ± 0.22 c	6.45 ± 0.71 c	77.10 ± 3.07 c	65.40 ± 2.47 c	31.26 ± 0.48 c	6408.10 ± 1149.57 c

**Note:** Values are means ± SE (*n* = 5). Different letters within the same year indicate significant differences among treatments (*p* < 0.05).

**Table 4:** Soil nutrient content in the 0–20 cm soil layer before sowing in 2023–2024.

Year	Total *p*	Total K	Total N	Available K	Available N	Available *p*	Organic Matter	pH
	(g/kg)	(g/kg)	(g/kg)	(mg/kg)	(mg/kg)	(mg/kg)	(g/kg)	
2023	0.63	6.01	0.51	75.8	38.26	41.98	8.79	8.76
2024	0.61	10.8	0.85	102.3	53.7	22.5	10.2	7.98

## Data Availability

The data that support this study are available upon reasonable request from the corresponding author. The data are not publicly available due to privacy and ethical restrictions.

## Supplementary Materials

The following supporting information can be downloaded at https://www.mdpi.com/article/10.3390/plants15081157/s1. Table S1: Comparison of sorghum plant height at different growth stages and under different treatments in 2023–2024; Table S2: Comparison of sorghum stem thickness at different growth stages and under different treatments in 2023–2024.
