# Supplementary material for: Film Mulching Drip Irrigation Improves the Soil Hydrothermal Environment to Enhance Photosynthetic Efficiency and Yield of Sorghum in an Agro-Pastoral Ecotone of Northern China
Source: Plants (Basel). 2026 Apr 9;15(8):1157. doi: 10.3390/plants15081157 (PMC13119227; doi:10.3390/plants15081157)
Supplement: Supplementary file 1 [file plants-15-01157-s001.zip › plants-4225323-supplementary.pdf]

Table S1. Comparison of sorghum plant height at different growth stages and under different treatments in 2023-2024

Year	Treatment code	Plant height (cm)			
		Jointing	Heading	Flowering	Filling
2023	FMDI	110.23±3.55a	199.15±6.21a	202.58±12.10a	202.25±5.77a
	FM	86.65±5.10c	161.45±7.67c	165.58±15.96b	154.23±4.46c
	DI	98.18±4.38b	186.40±3.86b	188.45±5.39a	195.40±2.27b
	CK	75.48±5.11d	150.33±8.62d	155.63±1.76c	148.30±3.27d
2024	FMDI	104.35±6.64a	166.20±5.12a	169.67±2.12a	167.43±5.94a
	FM	86.40±4.63b	107.13±11.94c	136.30±3.40c	144.53±2.45c
	DI	100.74±8.98a	153.88±2.66b	156.12±2.56b	158.35±1.60b
	CK	79.58±5.11b	109.13±8.62c	121.89±1.76d	124.30±3.27d

Note: FMDI: Film mulching drip irrigation, FM: Film mulching without drip irrigation, DI: Drip irrigation without film mulching, CK: Bare land without film mulching or drip irrigation. The value to the left of the ± sign represents the repeated measures mean (n=5), while the value to the right indicates the standard error. Different letters within the same year denote significant differences between treatments ($P < 0.05$).

Table S2. Comparison of sorghum stem thickness at different growth stages and under different treatments in 2023-2024

Year	Treatment code	Stem thickness (mm)			
		Jointing	Heading	Flowering	Filling
2023	FMDI	19.80±2.43a	22.43±2.79a	22.73±2.22a	21.98±4.21a
	FM	15.40±2.61b	17.73±2.59b	19.43±1.10bc	18.18±2.32ab
	DI	16.13±1.50b	19.00±1.27b	20.63±0.90ab	19.20±0.57ab
	CK	13.70±1.21b	16.60±1.40b	17.40±1.94c	16.73±2.29b
2024	FMDI	17.95±1.10a	23.85±1.11a	25.43±1.87a	25.70±2.45a
	FM	15.78±0.81b	17.60±1.07c	19.78±1.71b	19.60±1.06c
	DI	16.60±0.98ab	20.38±1.11b	20.56±1.62b	22.35±1.54b
	CK	13.88±1.47c	15.33±1.74d	16.28±1.21c	16.85±0.60d

Note: FMDI: Film mulching drip irrigation, FM: Film mulching without drip irrigation, DI: Drip irrigation without film mulching, CK: Bare land without film mulching or drip irrigation. The value to the left of the ± sign represents the repeated measures mean (n=5), while the value to the right indicates the standard error. Different letters within the same year denote significant differences between treatments ($P < 0.05$).
